# Impact of hemoglobin increase in patients with acute kidney injury and severe anemia on major adverse kidney events

**DOI:** 10.1080/0886022X.2026.2681245

**Published:** 2026-06-01

**Authors:** Guillermo Navarro-Blackaller, Jose J. Zaragoza, Fernando Cruz-Aragón, Daniela Ponce, Itzel Fong-Maravilla, Gael Chávez-Alonso, María F. García-Peña, Alejandro Martínez Gallardo-González, Eduardo Mendoza-Gaitán, Juan A. Gómez-Fregoso, Luz Alcantar-Vallin, Karina Renoirte-López, Ramón Medina-González, Manuel Arizaga-Nápoles, Guillermo García-García, Jonathan S. Chávez-Iñiguez

**Affiliations:** ^a^ Nephrology Service, Hospital Civil de Guadalajara Fray Antonio Alcalde, Guadalajara, Jalisco, Mexico; ^b^ University of Guadalajara Health Sciences Center, Guadalajara, Jalisco, Mexico; ^c^ Intensive Care Unit, Mexico Hospital H + Queretaro, Mexico; ^d^ Internal Medicine Department, Botucatu School of Medicine, University of Sao Paulo State, Sao Paulo, Brasil

**Keywords:** Acute kidney injury, anemia, hemoglobin correction, major adverse kidney events (MAKE), kidney replacement therapy

## Abstract

Acute kidney injury (AKI) is frequently accompanied by severe anemia, raising concerns that impaired oxygen delivery may accelerate kidney dysfunction. However, the clinical benefit of early hemoglobin (Hb) correction remains uncertain. This study evaluated the association between early Hb correction and major adverse kidney events (MAKE). We conducted a single-center retrospective cohort including hospitalized adults with AKI and baseline severe anemia (Hb <8.0 g/dL). Patients were stratified according to whether they achieved Hb correction (>10 g/dL) within the first 10 days of hospitalization. The primary outcome was MAKE at 10 days (MAKE10), defined as death, initiation of kidney replacement therapy (KRT), or ≥25% decline in estimated glomerular filtration rate (eGFR). Secondary outcomes included individual MAKE components and MAKE at 90 days (MAKE90). Multivariable logistic regression models were adjusted for age, sex, and baseline eGFR. Among 232 patients, 47 (20.3%) achieved Hb correction. Baseline characteristics were comparable between groups. Although unadjusted analyses suggested a lower incidence of MAKE10 in the correction group, adjusted analyses showed no significant association (adjusted OR 1.04; 95% CI 0.41–2.62). Hb correction was nor associated with each individual components of MAKE or MAKE90. No differences were observed according to the correction strategy (transfusion alone vs transfusion plus erythropoiesis-stimulating agents). In this retrospective cohort of patients with AKI and severe anemia, early Hb correction to >10 g/dL was not associated with improved short- or medium-term outcomes. These findings support a conservative and individualized approach to anemia management during AKI, although residual confounding and selection bias cannot be excluded.

## KEY LEARNING POINTS

**What was known:** Severe anemia is common in AKI and is associated with worse clinical outcomes, leading to the belief that early hemoglobin correction could improve tissue oxygen delivery and kidney recovery. However, robust evidence supporting aggressive hemoglobin normalization during AKI has been limited and inconsistent.

**This study adds:** In hospitalized patients with AKI and severe anemia, early hemoglobin correction to >10 g/dL was not associated with reduced MAKE, mortality, KRT initiation, or persistent kidney dysfunction. This lack of benefit was consistent regardless of whether correction was achieved with transfusion alone or combined with ESAs.

**Potential impact:** The findings support a conservative, individualized approach to anemia management during AKI and challenge the routine pursuit of aggressive hemoglobin normalization. Clinicians should avoid assuming that early correction of hemoglobin alone will improve kidney-related outcomes in this high-risk population.

## Background

In the setting of acute kidney injury (AKI), there are currently no therapies that directly target the kidney to restore its function [1]. Instead, clinical management focuses on treating metabolic complications and, in the most severe cases, providing kidney replacement therapy (KRT) [2]. Among these complications, clinical efforts are primarily directed toward correcting severe hyperkalemia, volume overload, metabolic acidosis, and uremia [3]. However, AKI is a syndrome of multiorgan dysfunction that promotes several metabolic abnormalities often overlooked in the acute setting, including hematologic alterations manifested as a reduction in hemoglobin (Hb) [4]. This complication may plausibly have a major impact on physiological processes and, consequently, on the clinical course of AKI [5]. A decline in Hb may compromise adequate tissue oxygenation and nutrient delivery, thereby hindering recovery during an episode of AKI, promoting maladaptive cellular responses, and favoring a pathogenic transition toward chronic kidney disease (CKD) [6,7]. Because an association has been documented between abrupt reductions in hemoglobin (Hb) and the risk of developing AKI [8], it is reasonable to consider that this relationship may also operate bidirectionally. In other words, increasing Hb levels during hospitalization for AKI could potentially be associated with improved clinical trajectories and lower rates of major adverse kidney events (MAKE). Although this association has been explored in other critical care contexts [9], its specific impact on kidney recovery in the setting of AKI remains largely unaddressed and subject to ongoing debate. Clarifying these relationships is clinically important because hemoglobin (Hb) is routinely measured, is potentially modifiable, and may serve as a pragmatic tool for risk stratification. Furthermore, given the distinct risks and mechanisms of action of available interventions, it is clinically relevant to examine whether the strategy used to increase Hb, either through blood transfusions and/or erythropoiesis-stimulating agents (ESAs), yields differential effects on kidney outcomes during AKI. Therefore, to address this critical knowledge gap and inform clinical practice, we investigated the association between early Hb correction in hospitalized patients with severe anemia and AKI and the occurrence of MAKE across short and medium term follow-up periods.

## Methods

This retrospective cohort study was conducted at Hospital Civil de Guadalajara Fray Antonio Alcalde, Mexico, a tertiary academic referral center. All hospitalized patients with AKI who received a nephrology consultation were screened for eligibility. We focused on a specific clinical phenotype consisting of patients with AKI and severe anemia at baseline, defined as a Hb concentration <8.0 g/dL. Eligibility further required at least three Hb measurements during follow-up to adequately characterize Hb trajectories, with the baseline value (<8.0 g/dL) compared with subsequent measurements. Hb levels were measured in the hospital’s certified central laboratory using the Cell-Dyn Ruby^®^ hematology analyzer.

Patients who achieved at least one Hb measurement >10 g/dL during follow-up were classified as the correction group, whereas those who did not were classified as the non-correction group. The Hb threshold of <8 g/dL was selected to identify severe anemia, whereas Hb correction >10 g/dL was considered a clinically meaningful increase beyond the severe anemia range. At least three Hb measurements were required to characterize Hb trajectories during follow-up, while correction was defined as the achievement of at least one Hb value >10 g/dL within the first 10 days of hospitalization. Hb target goals and decisions regarding intervention were highly individualized and based exclusively on the clinical judgment of the attending physicians and nephrology specialists. Anemia management strategies reflected real-world physician-directed practice and included red blood cell transfusion and, in selected patients, the use of erythropoiesis-stimulating agents (ESAs), specifically human recombinant erythropoietin (epoetin alfa) and erythropoietin analogs (darbepoetin). The diagnosis of AKI was established using serum creatinine (sCr) criteria as defined by the Kidney Disease: Improving Global Outcomes (KDIGO) guidelines [10]. For this study, patients were required to have a known baseline sCr level, defined as the most recent stable measurement obtained within six months prior to hospitalization. Availability of follow-up sCr measurements during subsequent hospital days was mandatory to evaluate the primary and secondary outcomes (MAKE10 and MAKE90). MAKE were defined as a composite of death, initiation of KRT, or *a* ≥ 25% decline in estimated glomerular filtration rate (eGFR) from baseline [11]. The MAKE10 and MAKE90 composite outcomes were selected to improve consistency and comparability of AKI-related endpoints across studies. In accordance with recommendations from the 31st Acute Disease Quality Initiative (ADQI) consensus, we identified the subphenotype of patients with AKI and severe anemia as a potential marker of response to transfusion and erythropoiesis-stimulating agent (ESA) therapy [1].

Patients were excluded if they had experienced AKI within the preceding three months, were younger than 18 years, had CKD stage 5, were receiving chronic dialysis, had undergone kidney transplantation, had a hospital stay shorter than 48 h, or had missing data that precluded complete analysis. Clinical characteristics, demographic information, and laboratory data were obtained through automated extraction from the institutional electronic medical record system. Indications for initiation of KRT included diuretic resistant fluid overload, severe hyperkalemia, severe metabolic acidosis, and uremic complications such as encephalopathy, pericarditis, or seizures [12].

### Study objectives

The primary objective was to evaluate the association between Hb correction (>10 g/dL within 10 days of hospitalization) and the occurrence of MAKE10 in hospitalized patients with AKI and severe anemia (Hb <8.0 g/dL). Secondary objectives were to assess the association between early Hb correction and the individual components of MAKE death, initiation of KRT, or *a* ≥ 25% decline in eGFR from baseline, as well as the association between Hb correction and the occurrence of MAKE90. An exploratory objective was to evaluate whether the strategy used to increase Hb, through blood transfusion alone or transfusion combined with ESAs, resulted in differential effects on kidney outcomes.

This study was funded by a grant from the Secretaría de Salud Jalisco and the Consejo Nacional de Ciencia, Humanidades y Tecnología (CONAHCYT) number SNI1. The requirement for written informed consent was waived by the Institutional Review Board (IRB) due to the retrospective nature of the study. The study was approved by the Hospital Civil de Guadalajara Fray Antonio Alcalde IRB (HCG/CEI-0550/15). All procedures were conducted in accordance with the Strengthening the Reporting of Observational Studies in Epidemiology (STROBE) guidelines [13] and the REporting of Studies Conducted using Observational Routinely Collected Health Data (RECORD) statement [14].

### Statistical analysis

Continuous variables were assessed for normality using the Shapiro–Wilk test and visual inspection of histograms. Given the non-normal distribution typical of critically ill populations, continuous variables are reported as medians with interquartile ranges (IQR). Categorical variables are expressed as absolute frequencies and percentages. Baseline characteristics between the exposure group (patients achieving Hb correction >10 g/dL within 10 days) and the non-correction group were compared using the Mann–Whitney U test for continuous variables and the chi-square test or Fisher’s exact test for categorical variables, as appropriate.

To assess the impact of Hb correction on the primary outcome, unadjusted risk ratios (RR) and absolute risk differences were calculated. For multivariable analysis, logistic regression models were constructed to estimate odds ratios (OR) with 95% confidence intervals (CI). The primary model for MAKE10 was adjusted *a priori* for clinically relevant confounders, including age, sex, and baseline estimated glomerular filtration rate (eGFR), calculated using the CKD-EPI 2021 equation. To minimize selection bias and preserve statistical power, variables with substantial missingness (e.g., admission lactate) were excluded from the final multivariable model, allowing inclusion of the entire cohort (*N* = 232) in the regression analysis.

Secondary analyses included: (1) separate logistic regression models for each individual component of MAKE10 and (2) assessment of long-term outcomes using MAKE90. As an exploratory analysis, we evaluated the differential impact of the hemoglobin correction strategy (blood transfusion alone versus ESAs. Hb trajectories over the first nine days of hospitalization were visualized using mean values with standard errors (SEM). A forest plot was generated to graphically summarize adjusted odds ratios across all primary and secondary outcomes.

All statistical analyses were performed using Stata software, version 16.1 (StataCorp, College Station, TX, USA). A two-tailed *p* value <0.05 was considered statistically significant.

## Results

**Figure 1.**
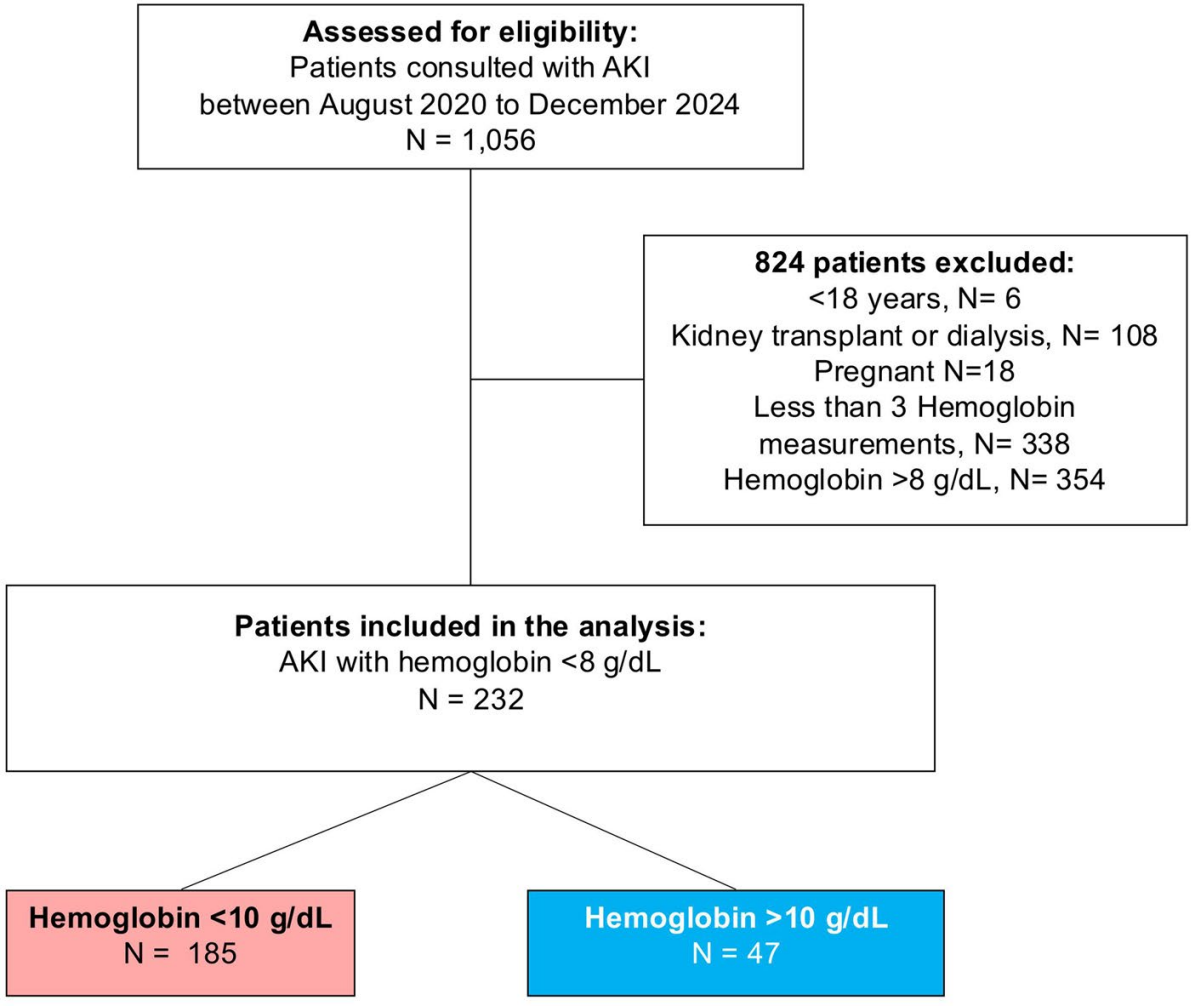
Study flow chart of the cohort. Flowchart illustrating the screening, exclusion, and final inclusion of patients with AKI and severe anemia. *Abbreviations:* AKI, Acute Kidney Injury; Hb, Hemoglobin.

Between August 2020 and December 2024, a total of 232 hospitalized patients with AKI and baseline Hb <8.0 g/dL were included in the final analysis. The cohort was stratified into two groups according to Hb correction (Hb >10 g/dL) status during the first 10 days of hospitalization. As shown in Figure 1, 47 patients (20.3%) achieved Hb correction, whereas 185 patients (79.7%) did not reach this target.

### Demographic and clinical characteristics

Baseline characteristics were well balanced between the two groups, indicating a homogeneous population at study entry, as shown in Table 1. The median age of the cohort was 49.0 years (IQR, 38.0–64.0), and 53.9% of patients were male. There were no significant differences in baseline sCr (0.90 [0.70–1.22] vs. 0.80 [0.70–1.00] mg/dL, *p* = 0.21) or median eGFR (100.6 [82.3–110.0] vs. 99.6 [91.9–105.3] mL/min/1.73 m^2^, *p* = 0.48) between groups. sCr at study inclusion was also similar between groups (4.68 vs. 4.09 mg/dL, *p* = 0.20), as was the distribution of KDIGO AKI stages (*p* = 0.49). Notably, KDIGO stage 3 predominated, accounting for 84.8% of the study population. Sepsis was the leading cause of AKI in both groups (45.0% vs. 36.2%; *p* = 0.32). Hypovolemia was significantly more frequent among patients who achieved hemoglobin correction (25.5% vs. 13.0%; *p* = 0.04), whereas cardiorenal, nephrotoxic, and post-surgical etiologies showed no significant differences between groups.

**Table 1.** Baseline demographic and clinical characteristics of the study population stratified by Hb correction.

Variable	No correction *n* = 185	Correction *n* = 47	Total *n* = 232	*p*
Age (y)	47.0 (38.0–64.0)	56.0 (40.0–64.0)	49.0 (38.0–64.0)	0.17
Male sex	101 (54.6%)	24 (51.1%)	125 (53.9%)	0.66
Baseline creatinine (mg/dL)	0.90 (0.70–1.22)	0.80 (0.70–1.00)	0.90 (0.70–1.20)	0.21
Baseline eGFR (ml/min/1.73 m^2^)	100.6 (82.3–110.0)	99.6 (91.9–105.3)	100.4 (85.5–109.5)	0.48
Creatinine at inclusion (mg/dL)	4.68 (2.80–6.81)	4.09 (2.33–5.70)	4.50 (2.71–6.70)	0.20
Stages of AKI				0.49
Stage 1	8 (4.3%)	3 (6.4%)	11 (4.7%)	
Stage 2	7 (3.8%)	3 (6.4%)	10 (4.3%)	
Stage 3	157 (84.9%)	40 (85.1%)	197 (84.9%)	
Hb (g/dL)	7.2 (6.5–7.6)	7.1 (6.3–7.7)	7.2 (6.5–7.6)	0.98
AKI etiologies				
Sepsis	83 (45%)	17 (36.2%)	100 (43.1%)	0.32
Hypovolemia	24 (13.0%)	12 (25.5%)	36 (15.5%)	0.04
Cardiorenal	19 (10.3%)	8 (17.0%)	27 (11.6%)	0.20
Nephrotoxic	34 (18.4%)	6 (12.8%)	40 (17.2%)	0.51
Post-surgical	25 (13.5%)	4 (8.5%)	29 (12.5%)	0.46
Leukocytes (/µL)	12.9 (8.4–18.1)	13.8 (8.3–19.4)	13.0 (8.3–18.2)	0.70
Platelets (/µL)	174 (88–304)	242 (122–322)	189 (93–314)	0.083
Potassium (mEq/L)	4.7 (4.1–5.5)	4.9 (4.2–5.9)	4.8 (4.1–5.6)	0.50
Sodium (mEq/L)	135 (130–140)	136 (131–140)	135 (130–140)	0.44
Bicarbonate (mEq/L)	17.9 (13.0–22.0)	19.1 (17.2–23.2)	18.3 (13.2–22.0)	0.31
pH	7.35 (7.28–7.41)	7.39 (7.33–7.41)	7.36 (7.30–7.41)	0.53
Lactate (µmol/L))	1.3 (0.8–2.9)	2.1 (0.8–3.5)	1.5 (0.8–3.0)	0.22
Urea (mg/dL)	163 (109–218)	137 (89–203)	158 (106–218)	0.11
Transfused	158 (85.4%)	46 (97.9%)	204 (87.9%)	0.019
Transfusions	5 (2–9)	5 (2–7)	5 (2–8)	0.52
Erythropoietin alfa	40 (21.6%)	9 (19.1%)	49 (21.1%)	0.71
Darbepoetin	7 (3.8%)	0 (0.0%)	7 (3.0%)	0.18
≥25% decline in eGFR from baseline)	140 (75.7%)	38 (80.9%)	178 (76.7%)	0.58
KRT	71 (38.4%)	19 (40.4%)	90 (38.8%)	0.80
Death	46 (24.9%)	8 (17.0%)	54 (23.3%)	0.35

Data are presented as median (interquartile range [IQR]) for continuous variables and frequency (percentage) for categorical variables. *P*-values were calculated using the Mann-Whitney U test for continuous data and the Chi-square test for categorical data. Hb Correction: Defined as achieving a hemoglobin level >10 g/dL within the first 10 days.

eGFR: Estimated Glomerular Filtration Rate calculated using the CKD-EPI 2021 creatinine equation. KRT: Kidney Replacement Therapy. Significant differences (*p* < 0.05).

Markers of critical illness severity at admission, including serum lactate (1.3 vs. 1.5 mmol/L, *p* = 0.22), were comparable between groups. Key metabolic parameters, including serum potassium, sodium, bicarbonate, pH, lactate, and urea, were also similar between groups (all *p* > 0.05). As expected given the stratification strategy, patients who achieved Hb correction had a higher frequency of blood transfusion compared with the non-correction group (97.9% vs. 85.4%, *p* = 0.019); however, the total number of transfused units did not differ between groups (median, 5 units; *p* = 0.52). The use of ESAs, including human recombinant erythropoietin, epoetin alfa (21.1%) and darbepoetin (3.0%), was similar between groups, with no statistically significant differences (all *p* > 0.05).

**Figure 2.**
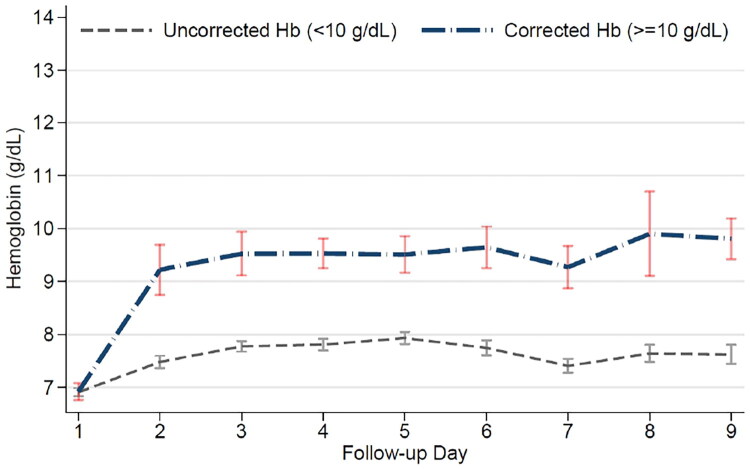
Temporal trajectory of mean hemoglobin levels during the first 10 days of follow-up. Comparison of daily hemoglobin concentrations (g/dL) between patients who achieved the correction target (>10 g/dL, blue line) and those who did not (gray dashed line). Data are presented as Mean Standard Error of the Mean (SEM). A clear separation between groups is observed from day 2 onwards. Note: Day 10 was excluded from the visualization to minimize variability due to discharge or censorship.

The difference in Hb levels between groups became evident from day 2 and was sustained throughout the follow-up period, as shown in Figure 2, confirming the effectiveness of the correction strategies employed. *A* ≥ 25% decline in eGFR from baseline occurred in 178 patients (76.7%), with no significant difference between groups (75.7% vs. 80.9%, *p* = 0.58). The proportion of patients requiring KRT was 90 (38.8%) and was nearly identical between groups (38.4% vs. 40.4%, *p* = 0.80). Overall, 54 patients (23.3%) died during follow-up, with similar mortality rates between groups (24.9% vs. 17.0%, *p* = 0.35).

### Primary outcome

In unadjusted bivariate analyses, achieving Hb levels >10 g/dL appeared to be strongly protective. The incidence of MAKE10 was significantly lower in the correction group (14.9%) compared with the non-correction group (93.5%), yielding a crude risk ratio of 0.16 (95% CI, 0.07–0.32; *p* < 0.001). However, this association was substantially attenuated after adjustment for confounders in the multivariable logistic regression model (Table 2). After controlling for age, sex, and baseline eGFR in the full cohort (*N* = 232), Hb correction was no longer independently associated with a reduction in MAKE10 (adjusted OR, 1.04; 95% CI, 0.41–2.62; *p* = 0.93). This discrepancy between crude and adjusted estimates suggests that the apparent unadjusted benefit may have been driven by underlying clinical characteristics or physiological reserve that favored patients more likely to respond to hematologic interventions, rather than Hb correction itself being the primary determinant of kidney recovery.

**Table 2.** Unadjusted and multivariate logistic regression analysis of the association between hemoglobin correction and clinical outcomes.

	Unadjusted analysis			Adjusted analysis		
Primary outcome						
	OR	95% CI	*P*-value	OR	95% CI	*P*-value
MAKE10	1.15	[0.47–2.80]	0.758	1.04	[0.41–2.62]	0.931
Secondary outcomes						
Mortality	0.68	[0.29- .56]	0.360	0.59	[0.25–1.40]	0.232
New KRT Requirement	1.09	[0.57–2.09]	0.797	1.17	[0.60–2.31]	0.642
Decrease in eGFR >25%	1.36	[0.61–3.02]	0.455	1.22	[0.52–2.86]	0.644
MAKE90	0.83	[0.40–1.72]	0.617	0.86	[0.41–1.80]	0.684

Crude OR: Unadjusted bivariate association.

Adjusted OR: Multivariate model adjusted for age, sex, and baseline eGFR.

MAKE10: Composite outcome of death, new requirement for KRT, or persistent renal dysfunction (defined as a decrease in eGFR >25% from baseline) at 10 days or hospital discharge. MAKE90: Composite outcome assessed at 90 days. CI: Confidence Interval; OR: Odds Ratio.

### Secondary outcomes

**Figure 3.**
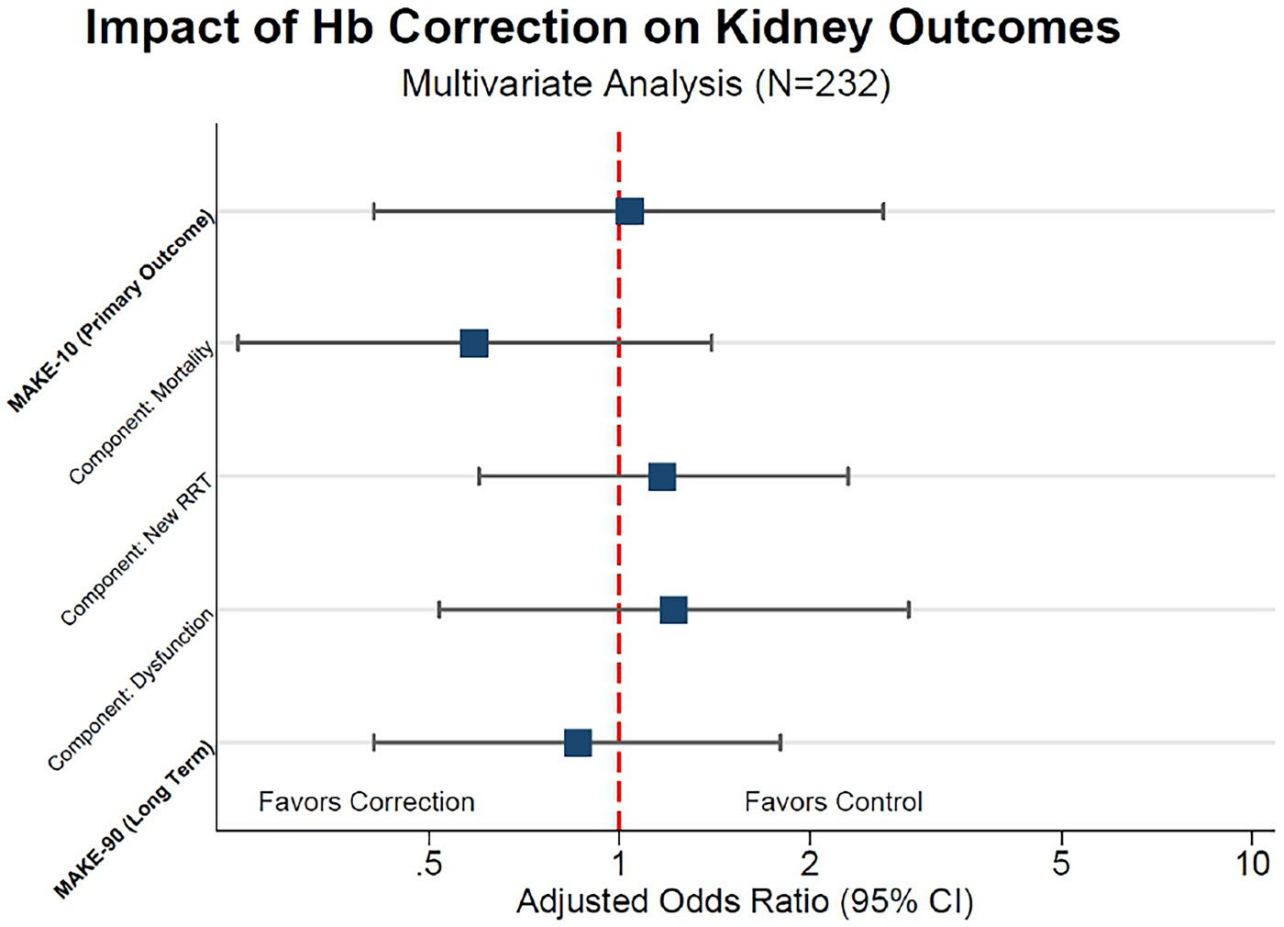
Forest plot of adjusted odds ratios for primary and secondary kidney outcomes. Visual summary of the multivariate logistic regression analysis (*N* = 232). The markers represent the Adjusted Odds Ratio (aOR) for the effect of achieving Hb correction >10 g/dL on each outcome. Horizontal bars indicate 95% Confidence Intervals (95% CI). Primary Outcome: MAKE10. Secondary Outcomes: Individual components of MAKE10 (Mortality, New RRT, ≥25% decline in eGFR from baseline) and MAKE90. Model Adjustment; All models were adjusted for age, sex, and baseline eGFR (CKD-EPI 2021). The vertical dashed line at 1.0 represents the line of no effect. Values to the left favor hemoglobin correction. Abbreviations: RRT, Renal Replacement Therapy; ESA, Erythropoiesis-Stimulating Agents.

We further analyzed the individual components of the composite MAKE10 outcome to identify potential signals of benefit or harm (Table 2 and Figure 3). Hb correction was not independently associated with reduced mortality (adjusted OR, 0.59; 95% CI, 0.25–1.40; *p* = 0.23). Similarly, the risk of requiring new KRT (adjusted OR, 1.17; 95% CI, 0.60–2.31) and the likelihood of an eGFR decline >25% from baseline (adjusted OR, 1.22; 95% CI, 0.52–2.86) did not differ significantly between groups. Medium-term outcomes were consistent with these short-term findings, as no significant association was observed between early Hb correction and the composite MAKE90 outcome (adjusted OR, 0.86; 95% CI, 0.41–1.80; *p* = 0.68).

In exploratory multivariable analyses stratified by anemia correction strategy (blood transfusion alone versus transfusion plus ESAs), neither approach was independently associated with improved MAKE outcomes. This consistency across treatment modalities reinforces the interpretation that attainment of an Hb target >10 g/dL, irrespective of the method used, likely reflects the underlying clinical trajectory rather than a direct independent therapeutic effect on kidney outcomes.

## Discussion

**Figure 4.**
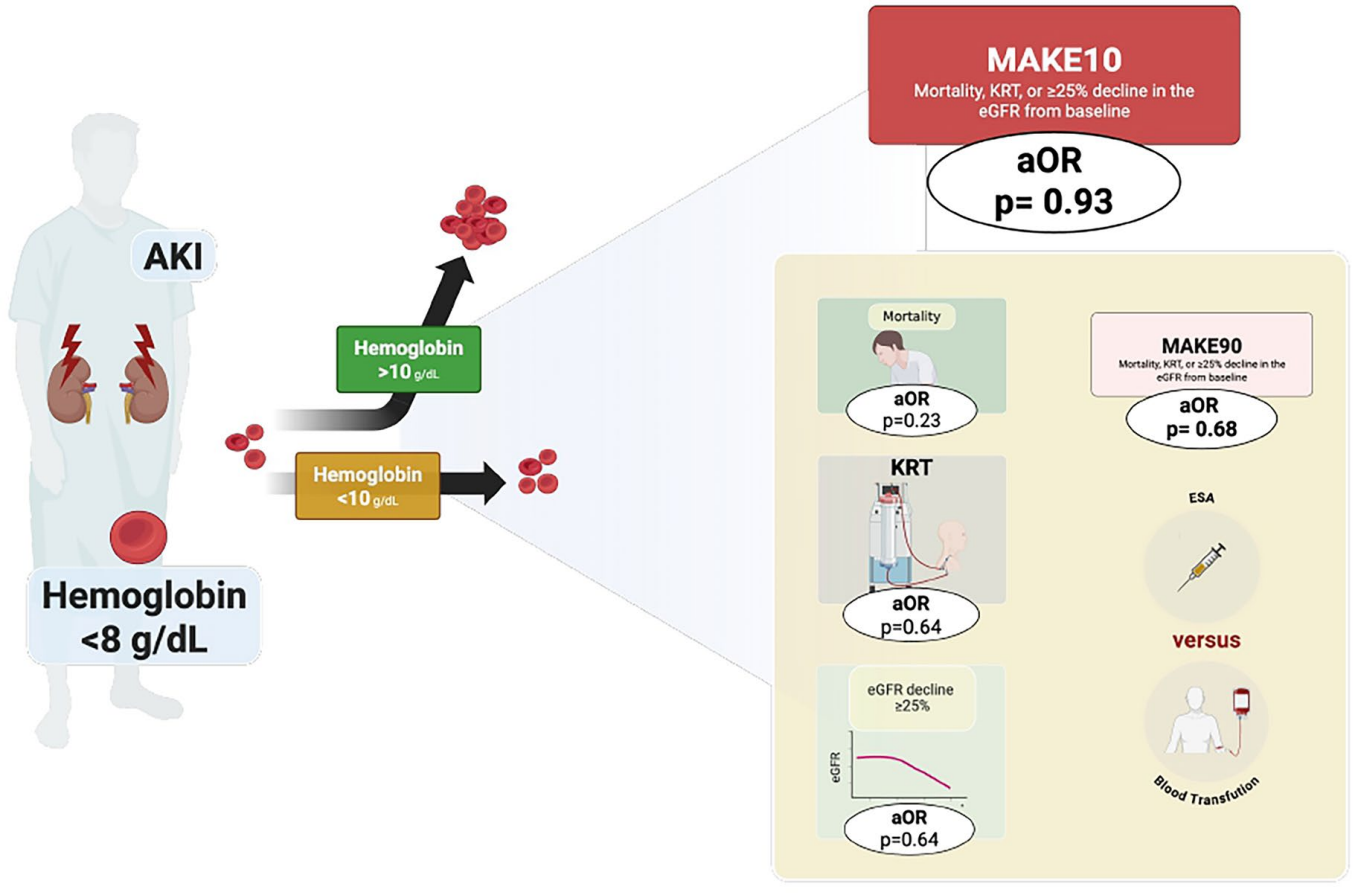
Central image.

In patients with AKI and severe anemia, achieving an increase in Hb to >10 g/dL within the first 10 days of hospitalization was not associated with improved short- or medium-term MAKE, nor with any of the individual components of this composite outcome. Furthermore, no differential effect was observed according to the strategy used to increase Hb levels, whether through blood transfusion alone or transfusion combined with ESAs (Figure 4).

Despite early correction of Hb to >10 g/dL, no improvement in MAKE was observed. This lack of benefit is consistent with the limited, though growing, body of evidence suggesting that aggressive anemia management may not improve kidney outcomes in AKI. In a retrospective cohort of 212 patients, Hu et al. evaluated whether anemia was associated with lower rates of kidney recovery and poorer survival among individuals with AKI by comparing patients with mild versus severe anemia. In their study, neither kidney recovery nor survival differed between groups, regardless of whether anemia severity was defined by an Hb decline >2 versus ≤2 g/dL or by a nadir Hb >9 versus ≤9 g/dL (*p* > 0.5 for both) [4]. It is important to note that the characteristics of our cohort differ substantially from those of the aforementioned study, as we specifically focused on patients with AKI and severe anemia, defined as Hb <8 g/dL. This represents a more vulnerable clinical phenotype, potentially more susceptible to AKI-related complications and, therefore, theoretically more likely to benefit from Hb correction. In our cohort of patients with AKI and severe anemia, only approximately 20% achieved an Hb level >10 g/dL during hospitalization.

Although the composite MAKE outcome was not significantly associated with Hb correction, both mortality and KRT initiation remained frequent across groups, reflecting the high severity of illness of this cohort. These findings suggest that, in patients with severe AKI and profound anemia, early Hb correction alone may be insufficient to substantially modify short-term kidney-related outcomes. The marked attenuation of the unadjusted association after multivariable adjustment suggests that baseline clinical differences and residual confounding likely contributed to the crude differences observed between groups.

This lack of a statistically significant association is particularly noteworthy, given that our initial hypothesis was strongly supported by established pathophysiological mechanisms. At the cellular level, severe anemia may impair tubular epithelial cell and macrophage function, thereby promoting inflammation and fibrosis, whereas improvement in Hb levels has been shown to mitigate these abnormalities and enhance both structural and functional renal parameters [15]. In addition, severe anemia may increase susceptibility to further tubular injury; thus, restoration of Hb to a near-normal range could theoretically reestablish intracellular homeostasis and confer protection against ongoing renal damage [16]. Based on this robust physiological evidence, we hypothesized that, in the setting of AKI and severe anemia, improvement in Hb levels might also confer a medium-term survival benefit, potentially independent of its effects on kidney function. Consistent benefits related to kidney physiology have been described when severe anemia is corrected, including prevention of the transition from AKI to CKD [17], enhanced tissue oxygenation [18], improved nephron metabolism [18], preservation of kidney function [19], and improved quality of life [20].

In our study, nearly nine out of ten patients received at least one blood transfusion during hospitalization, with transfusions occurring more frequently in the correction group (*p* = 0.019). Despite this, no clinical benefit was observed with respect to MAKE outcomes. Blood transfusion remains common in critically ill patients, although its use has gradually declined with the adoption of more restrictive transfusion strategies [21]. Because nearly all patients in the correction group received blood transfusions, it is possible that transfusion-related adverse effects, including volume overload, inflammatory responses, and transfusion-related immunomodulation, may have partially offset the potential physiological benefits of hemoglobin correction, contributing to the neutral outcomes observed. The absence of benefit in MAKE outcomes among patients with AKI and severe anemia, despite receiving a median of five units of packed red blood cells, is consistent with findings from a meta-analysis of 31 clinical trials, which demonstrated no mortality benefit when comparing liberal versus restrictive transfusion strategies, the latter typically defined by transfusion thresholds of hemoglobin levels below 7 g/dL [22,23]. A cochrane review including 48 clinical trials found that 30-day mortality was not affected by adopting a restrictive transfusion strategy, defined as transfusion at Hb <8 g/dL, compared with a liberal strategy (<9 g/dL); however, MAKE outcomes were not evaluated in that analysis [23]. Restrictive transfusion strategies have also been examined in critically ill populations with a specific focus on renal outcomes, and similarly, no favorable effects have been demonstrated [24]. Importantly, restrictive approaches reduce the likelihood of receiving a red blood cell transfusion by approximately 40% [23]. Notably, current American College of Chest Physicians guidelines consider certain comorbidities on an individual basis when issuing recommendations on liberal versus restrictive transfusion thresholds, yet provide no specific guidance regarding transfusion practices in the setting of AKI [24]. Studies such as ours may help address this gap in the evidence.

During AKI, increased erythropoietin (EPO) production has been observed across multiple organs, an effect that appears to occur independently of oxygen tension [25]. In parallel, EPO receptors are upregulated within the kidney parenchyma [26], suggesting that activation of these receptors may promote cellular repair processes [27,28]. Experimental studies have demonstrated that administration of high-dose EPO attenuates AKI severity [29–31]. Accordingly, it was biologically plausible to hypothesize that increasing Hb levels during AKI through EPO administration could confer clinical benefit. For this reason, we performed an exploratory analysis examining the use of ESAs. Despite ESA administration, Hb trajectories did not differ between groups, and no clinical benefit was observed among patients who received EPO. Importantly, current KDIGO anemia guidelines do not support ESA use for rapid correction of anemia during acute illness, and the delayed hematologic response of ESAs likely limits their short-term impact during AKI hospitalization.

These findings are consistent with the lack of effect reported in prior clinical trials, including EPO-TBI [32] and EAKI [33]. This observation may reflect a state of ESA resistance. Although the mechanisms underlying ESA hyporesponsiveness in AKI remain incompletely understood, prior studies suggest that poor Hb responsiveness to high doses of EPO may promote endothelial dysfunction and reduced nitric oxide bioavailability, thereby increasing cardiovascular risk [18]. Alternatively, this pattern may reflect a more severely ill and pro-inflammatory patient population.

Our findings contribute to the ongoing clinical debate regarding aggressive anemia correction in AKI. Specifically, our results are consistent with the conservative approach recommended by current KDIGO guidelines, which caution against routine ‘normalization’ of Hb levels in patients with AKI, particularly when targeting values above 10 g/dL [10]. The narrow therapeutic window between potential benefits, such as avoidance of KRT, reduced transfusion requirements, and alleviation of anemia-related symptoms; and potential harms, including cardiovascular events and increased mortality, underscores the importance of individualized decision-making when prescribing ESAs.

The strengths of our study include the uniqueness of the cohort, which comprised patients with AKI and severe anemia, and its direct relevance to everyday clinical practice. We applied robust statistical methods with multivariable adjustment and relied on a validated clinical database with clearly defined outcomes.

Nevertheless, these strengths should be interpreted in light of several inherent methodological limitations. A post-hoc power analysis for MAKE10, based on the observed group sizes and effect estimate, showed limited statistical power (approximately 6.1%), indicating that the study was underpowered to detect small differences between groups; therefore, a Type II error cannot be excluded. The retrospective observational design allows for the identification of associations but precludes causal inference. The relatively small number of patients achieving Hb correction limits statistical power, the robustness of multivariable analyses, and the generalizability of our findings. Although daily Hb values were available, the exact timing of MAKE components was not consistently recorded; therefore, Hb correction could not be modeled as a time-dependent exposure. Baseline kidney function was defined using the most recent stable sCr value within 6 months before hospitalization, which may not fully reflect kidney function immediately prior to AKI onset. Because most patients had KDIGO stage 3 AKI, the findings primarily reflect a population with severe kidney injury and may not be generalizable to patients with milder AKI (stages 1–2). The relatively small number of patients with less severe AKI precluded robust subgroup analyses according to AKI stage. Because the cohort included only patients who received nephrology consultation, the study may be subject to severe-case selection bias, limiting generalizability to patients with milder AKI managed without nephrology involvement. The definition of severe anemia (Hb <8 g/dL), the target hemoglobin level used to define correction (>10 g/dL), and the timing of anemia diagnosis in relation to AKI onset were arbitrarily chosen. Pre-hospitalization hemoglobin values were not consistently available, limiting evaluation of the magnitude and chronicity of anemia before AKI onset. Because hemoglobin correction strategies were based on physician clinical judgment rather than a standardized protocol, confounding by indication and residual selection bias cannot be excluded. It is possible that some patients in the non-correction group were perceived as either too critically ill or clinically stable to warrant aggressive hemoglobin correction, which may have introduced treatment-selection bias. Because Hb correction was defined as any value >10 g/dL within the first 10 days, temporal overlap between exposure and MAKE10 assessment cannot be completely excluded. The specific etiology of anemia could not be consistently determined. Information regarding active bleeding, recent surgical procedures, or invasive interventions was not consistently available, limiting evaluation of the potential impact of ongoing blood loss on treatment response and outcomes.

The absence of detailed hematologic and inflammatory biomarkers limited characterization of anemia phenotypes and precluded subgroup analyses according to specific anemia mechanisms. The retrospective design prevented reliable differentiation between isolated ESA therapy and combined transfusion plus ESA treatment. Because detailed ESA dosing data and iron/inflammatory biomarkers were not consistently available, the potential influence of inflammation and iron-restricted erythropoiesis on ESA responsiveness could not be adequately evaluated. Additionally, we lacked access to ancillary parameters that could further characterize anemia, such as iron deficiency status and bleeding events. These omissions may have confounded the assessment of anemia etiology, management, and prognosis. We were unable to formally evaluate the presence of EPO resistance due to insufficient data, which may have influenced the effectiveness of ESA therapy. Decisions regarding blood transfusion or ESA use were based on clinical judgment, introducing the possibility of unmeasured confounding inherent to non-randomized study designs. Detailed information regarding ESA dose, timing, duration, and administration frequency was not consistently available, limiting interpretation of the exploratory analyses involving ESA therapy. Although we adjusted for key covariates, residual confounding cannot be excluded. Finally, long-term follow-up data related to blood transfusions may be incomplete. Although institutional policy recommends transfusion at Hb levels <7 g/dL, consistent with the cohort’s median baseline Hb of 7.2 g/dL (IQR, 6.5–7.6), detailed documentation of all transfusion events during the 90-day follow-up period may have been subject to incomplete recording.

From a clinical perspective, our study provides valuable real-world evidence to inform therapeutic decision-making in a highly vulnerable population, patients with AKI and severe anemia, in whom uncertainty regarding Hb correction strategies remains substantial. By demonstrating that early Hb correction to levels >10 g/dL does not translate into a reduction in MAKE, our findings support a more conservative and individualized approach to anemia management during AKI. Such an approach may help reduce unnecessary transfusions and avoid exposure to ineffective or potentially burdensome interventions. Importantly, this study challenges long-standing assumptions regarding the benefits of Hb normalization in acute illness and highlights that Hb correction alone should not be pursued as a surrogate marker of kidney recovery. These insights may contribute to refining clinical protocols, optimizing resource utilization, and guiding future prospective studies aimed at identifying patient subgroups that may truly benefit from targeted Hb based interventions

## Conclusions

In this retrospective cohort of patients with AKI and severe anemia, correction of Hb levels to >10 g/dL, compared with failure to achieve this target, was not associated with short- or medium-term MAKE.

## Data Availability

The files and data are in the physical and electronic archives of the Civil Hospital of Guadalajara Fray Antonio Alcalde and can be requested with prior authorization. All data generated or analyzed during this study are included in this article. Further inquiries can be directed to the corresponding author
